# Deciphering Molecular Embeddings with Centered Kernel Alignment

**DOI:** 10.1021/acs.jcim.4c00837

**Published:** 2024-09-25

**Authors:** Matthias Welsch, Steffen Hirte, Johannes Kirchmair

**Affiliations:** † Department of Pharmaceutical Sciences, Division of Pharmaceutical Chemistry, Faculty of Life Sciences, University of Vienna, Josef-Holaubek-Platz 2, Vienna 1090, Austria; ‡ Christian Doppler Laboratory for Molecular Informatics in the Biosciences, Department for Pharmaceutical Sciences, University of Vienna, Vienna 1090, Austria; § Vienna Doctoral School of Pharmaceutical, Nutritional and Sport Sciences (PhaNuSpo), University of Vienna, Vienna 1090, Austria

## Abstract

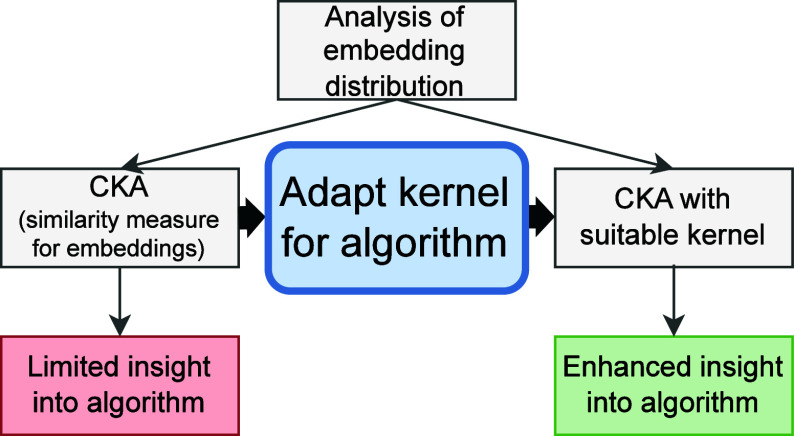

Analyzing machine learning models, especially nonlinear ones, poses significant challenges. In this context, centered kernel alignment (CKA) has emerged as a promising model analysis tool that assesses the similarity between two embeddings. CKA’s efficacy depends on selecting a kernel that adequately captures the underlying properties of the compared models. The model analysis tool was designed for neural networks (NNs) with their invariance to data rotation in mind and has been successfully employed in various scientific domains. However, CKA has rarely been adopted in cheminformatics, partly because of the popularity of the random forest (RF) machine learning algorithm, which is not rotationally invariant. In this work, we present the adaptation of CKA that builds on the RF kernel to match the properties of RF. As part of the method validation, we show that the model analysis method is well-correlated with the prediction similarity of RF models. Furthermore, we demonstrate how CKA with the RF kernel can be utilized to analyze and explain the behavior of RF models derived from molecular and rooted fingerprints.

## 1 Introduction

*In silico* models for predicting the physicochemical, biological, and structural properties of compounds can substantially enhance efficiency in drug discovery and related fields. For example, these models offer guidance on optimizing experimental resources and prioritizing compound synthesis and evaluation.^1,2^ For building *in silico* models, data representation in a machine-readable format is a prerequisite. Molecules, for example, can be represented by their physicochemical properties,^3^ topological fingerprints,^4,5^ or molecular graphs.^5,6^

Historically, simple linear models were used for assessing and predicting molecular properties. Although globally interpretable, their applicability is limited due to low expressive power. Today, machine learning methods like k-nearest neighbor (kNN) approaches, random forests (RFs), support vector machines (SVMs), and neural networks (NNs) prevail in molecular property prediction.^7−9^ While all are nonlinear, their properties and behavior can vary, e.g., regarding the scale of the feature matrix. Consider, e.g., using a feature matrix derived from the number of heavy atoms and molecular weight of molecules to generate a kNN model. The molecular weight will dominate the prediction of this model because of its larger magnitude. Models can also differ concerning the sensitivity toward rotations of the feature matrix.^10^ When the Euclidean distance is used, a kNN model will remain unaffected by data rotation because the Euclidean distance is preserved under rotation. However, when the Manhattan distance is used, the model will be sensitive to data rotation.

NNs have garnered significant interest due to their versatility and performance. They can process nonvector data representations and learn internal representations (embeddings), leading to high performance, particularly in data-limited regimes.^11^ Convolutional neural networks (CNNs), for example, reduce the number of connections between layers by employing learnable weights within sliding filters applied to images.^12,13^ Similarly, graph neural networks (GNNs) process graphs in a permutation-invariant manner.^6,14^ Additionally, there has been a surge in the popularity of models capable of handling sequence data, fueled by recent advancements in natural language processing.^15−17^

As the complexity of models increases, so do the challenges involved in analyzing models. However, model analysis is essential to the development of effective models. One type of tool for investigating and optimizing the inner workings of machine learning models is measures for the similarity of embedding distributions. For example, these tools have been used to show that a block structure of representational similarity emerges in NNs that originates primarily from the propagation of the first principal component. Some layers in this block of high representational similarity can be removed with minimal performance decrease, making inference cheaper and faster.^18,19^

Similarity measures between embedding distributions can also be used to show that a model exhibits a desirable property. For instance, they have been employed to assess whether models, expected to be robust to shifts in data distribution, remain stable under such shifts.^20^

Models learning similar representations are expected to produce similar outputs. Therefore, methods for comparing two embeddings derived from the same data set should correlate embedding similarity with performance similarity.^21−23^ Most of the existing methods for embedding similarity comparison either aim to find a correlation between the embeddings^21,24−26^ or compare the manifolds on which the data points lie.^27,28^ The latter type of method is based on the manifold hypothesis that high-dimensional real-world data points lie on a comparatively low-dimensional manifold.^29−31^

Historically, canonical correlation analysis (CCA) was used to infer information from the cross-covariance between two sets of random variables.^24^ However, CCA is sensitive to perturbations if the embedding matrices are not well conditioned.^32^ This limitation has been addressed with singular-vectors canonical-correlation analysis (SVCCA), which performs CCA on representations of truncated singular value decompositions of the embeddings.^25^ Another approach to limit the sensitivity of CCA to changes in the input is projection-weighted CCA (PWCCA). PWCCA replaces the mean in CCA with a weighted mean.^26^ It is also possible to compare embeddings by finding the minimal rotation from one embedding projected on the unit ball to the other. This problem is known as the orthogonal Procrustes problem.^23,33,34^

There are also topological methods for comparing embeddings, such as the Intrinsic Multiscale Distance (IMD). IMD estimates a lower bound for the spectral Gromov-Wasserstein distance between two data manifolds by analyzing the heat kernel traces of Laplacians from k-nearest neighbor graphs constructed from feature matrices.^27^ Representation Topology Divergence (RTD) compares two embeddings by considering each representation as a graph, with nodes being samples and the edges containing the distance between two samples. RTD evaluates the disparity in the topology of these graphs to quantify the differences between embeddings.^28^

Another method for comparing embeddings is centered kernel alignment (CKA). CKA was designed to fulfill the intrinsic properties of NNs, such as invariance to orthogonal transformation.^21^ The method is based on the Hilbert–Schmidt Independence Criterion (HSIC), which tests whether two sets of variables are independent.^21,35^ Calculating the CKA with the linear kernel is magnitudes faster than RTD and IMD.^28^ CKA excels at correlating layers of two networks with the same architecture but different training seeds.^23^

CKA has been successfully employed in various scientific domains.^36^ For example, in natural language processing, CKA revealed that the fine-tuning of models mainly affects the late layers of the NNs.^37^ In computer vision, CKA was employed to demonstrate that the removal of skip connections can lead to changes in the representation structure of vision transformers, causing a drop in model performance.^22^ CKA was also used to determine the robustness of models to graph size shifts by comparing node embeddings of original and coarsened graphs.^20^

Applications of CKA have been reported in various scientific domains.^36^ In cheminformatics, its adoption remains in the early stages,^20^ one reason being the popularity of RFs in this field. In contrast to NNs, for which CKA was designed, RFs are not rotationally invariant, and their embeddings are not readily accessible. Thus, adaptations of CKA are required to match the characteristic properties of RFs.

This work introduces and validates the adaptations required for using CKA with RFs. More specifically, we combine the RF kernel with CKA to obtain the internal representations of RFs. This matches the sensitivity of the similarity measure (CKA with RF kernel) and the learning algorithm (RF) toward data rotation. We verify that matching the behavior of the model and the kernel for CKA concerning data rotation is important by showing that convolutional encoders can lead to rotated embeddings. To validate that CKA with the RF kernel is a good similarity measure, we show its high correlation with model prediction similarity, thus reinforcing the RF kernel as suitable for RFs. Furthermore, we demonstrate the utility of the novel model analysis tool by utilizing it to (i) explain why the optimization of fingerprint lengths does not yield substantial changes in prediction performance and (ii) gain an understanding of models derived from topological fingerprints for the prediction of atom-based properties.

## 2 Methods

### 2.1 Rotationally Invariant Learning Algorithms

Let *M* ∈ *SO*(*p*) be a rotational matrix and  be a feature vector. Consider  to be a training set and  to be the training set rotated by *M*. Further, let *L* be a deterministic learning algorithm with the prediction *L*[*S*](*x*) when trained on *S* and predicting *x*.

### Definition 1 (A. Ng^10^)

A learning algorithm *L* is called rotationally invariant if, for any training set *S*, rotational matrix *M*, and test example *x*, *L*[*S*](*x*) = *L*[*MS*](*Mx*) holds.

**Figure 1:**
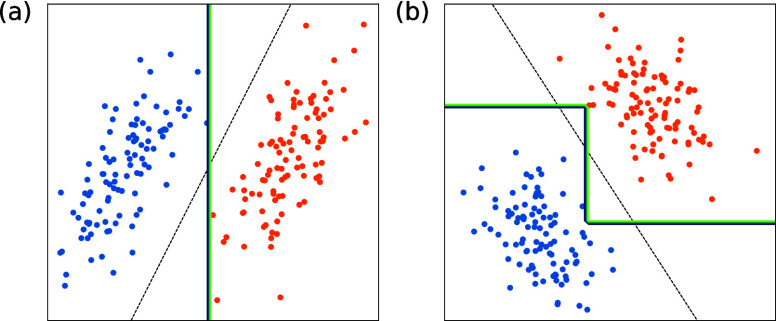
Two-dimensional classification problem addressed by an algorithm that is rotation-invariant (logistic regression) and one that is not rotation-invariant (decision tree). (a) Nonrotated data and (b) data rotated by π/3. The decision boundaries are indicated as black dashed lines for the logistic regression and as green continuous lines for the decision tree. Notably, the decision boundary of the logistic regression is rotated by π/3 while the decision tree’s boundary is completely different.

Examples of rotationally invariant algorithms include logistic regression and MLPs trained using back-propagation (provided that the weights are initialized using independent samples from a spherically symmetric distribution).^10^ In contrast, decision trees (which perform feature-axis aligned splits) and, hence, RFs^10,38^ are examples of classifiers that are not rotationally invariant.^10^ This behavior can be observed in practice when rotating tabular data: An RF classifier’s performance on test data will drop substantially while the performance of a NN will not be affected.^38^Figure 1 illustrates this behavior for logistic regression and a decision tree using synthetic data. Panel (a) of this figure shows an example of classification for nonrotated data, while panel (b) illustrates this data rotated by π/3. While the decision boundary for the decision tree classifier is entirely different, the decision boundary for the rotation invariant logistic regression is rotated by the same amount. This behavior should be reflected by similarity indices used for comparing embeddings with respect to a particular model.

### 2.2 Centered Kernel Alignment

CKA is a similarity index for two matrices,  and , which can be used to compare the representations of *n* samples even when their dimensionalities *p*_1_ and *p*_2_ are different. The CKA with the kernels *κ*(·,·) and  is defined as
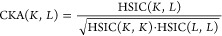
1with  and  being entries of the Gram matrices. The HSIC measures independence between two distributions. Its empirical estimator is
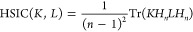
2with *H*_*n*_ being the corresponding centering matrix . The estimator converges to the population value with . However, the HSIC is not invariant to isotropic scaling. It is, therefore, normalized to yield the isotropically invariant CKA. CKA is in the interval [0,1], where 1 indicates that the representations are strongly dependent while 0 indicates independence.^21,35^ CKA depends on both the data set and the embedding strategy used. In a typical experiment, either the data set or the embedding strategy remains fixed while the other is changed to assess similarity. Afterward, a comparison is made between the similarities. A typical benchmark for what is considered high similarity is approximately 0.7. However, the threshold is data-dependent. Hence, comparisons across random seeds are performed to give a reference value.^20,21^ Linear CKA is invariant to isotropic scaling and orthogonal transformation because of the normalization and the dot product kernel, respectively, but not to a general invertible linear transformation.^21,35^ We used the implementation of CKA from ref. (39).

### 2.3 Random Forest Kernel for Centered Kernel Alignment

**Figure 2:**
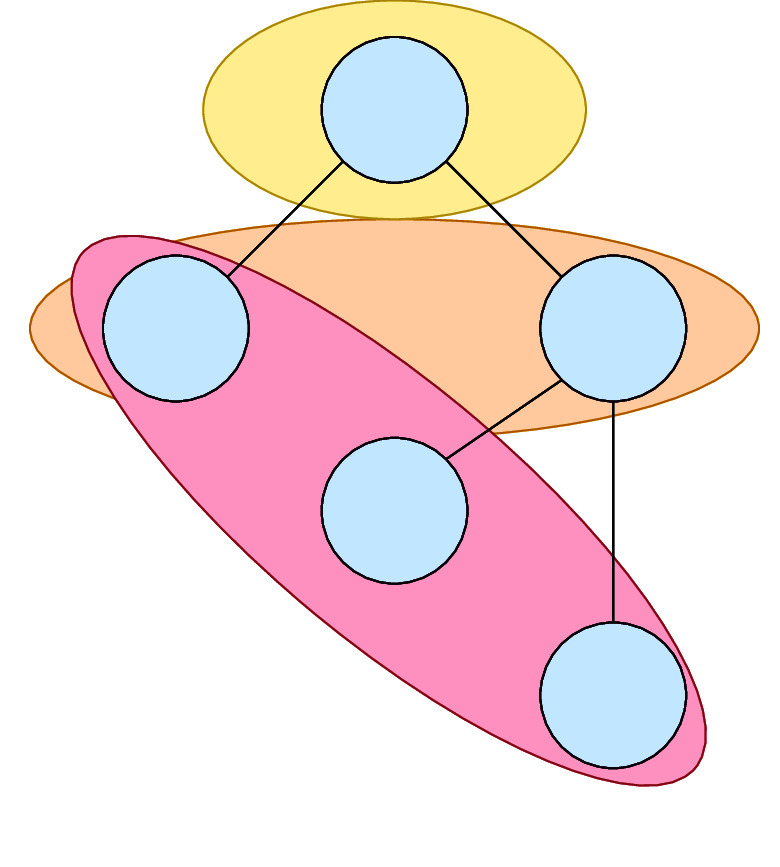
Decision tree dividing the data set into three partitions.


Every partitioning distribution  induces the following kernel for the data set *S*:
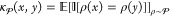
3where  is the indicator function, yielding 1 if *x*,*y* ∈ *S* are in the same block of a partition ρ and else 0.^40^ A RF can be viewed as a partitioning distribution, which partitions the data along the height of its trees. At the root of every decision tree, every element in *S* is part of the same block.^40^ Therefore, all combinations of two data instances share the block in that partition. Figure 2 shows the other partitions generated by one decision tree. It is possible to sample from this distribution by randomly selecting trees and depths and then checking if two data points share the same block.^40^ An overview of how CKA is combined with the RF kernel (CKA_rf_) is presented in Algorithm 1. We draw 500 sample partitions to calculate the RF kernel.

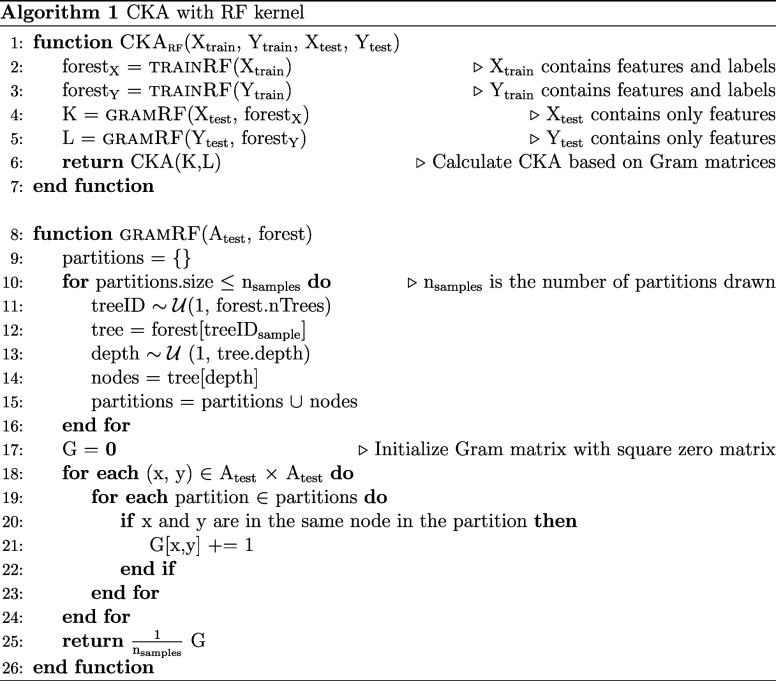
3a

### 2.4 Convolutional Neural Networks Can Create Rotated Embeddings

MNIST was selected to show that rotated embeddings can occur after convolutional encoders because the data set is simple and has multiple representations for each image. Three representations of the MNIST^41^ data set of handwritten digits were considered: the images,^41^ grid-like graphs constructed from the images (MNISTSuperpixel^42^), and sequences from the order of pixels that are drawn (MNISTStroke^43^). In this study, the task of all data sets was to identify digits below five as the positive class. For the data sets, a CNN, a GNN, and an LSTM were used as convolutional encoders, respectively.

#### 2.4.1 ResNet for MNIST

The images were normalized, and 50 000 were used for training. The images were initially fed through a convolutional layer with 64 channels, a kernel of size 7 × 7 with a stride of 2, and a padding of 3. Then, the ResNet18^12^ architecture was used up to the average pooling operation because the images are grayscale. On top of this convolutional encoder, an MLP with batch normalization, ReLU as the activation function, 64 hidden dimensions, and 2 layers were used for classification. RMSProp was employed as an optimizer, with a learning rate of 0.005 and a batch size of 256. The other parameters of RMSProp were chosen as the default implementation of torch.^44^

#### 2.4.2 GNN for MNISTSuperpixel

The coordinates of the nodes were added as node features. The message-passing base consisted of 4 layers of GINs with two internal layers each. Batch normalization was employed, and the number of node features was 32. ADD pooling was used as the readout operation, and the MLP following the GNN consisted of 2 layers with a hidden dimensionality of 64. Adam was used to train the network with a learning rate of 0.001 and a weight decay of 5 × 10^–4^ and otherwise default parameters. The batch size was set to 256. The other parameters were chosen as the default torchGeometric.^45^

#### 2.4.3 LSTM for MNISTStroke

The MNISTStroke data set was downloaded from ref. (43) and the loader was obtained from ref. (46). An LSTM with a hidden state of size 32 was used as an encoder. Its outputs were summed and fed through an MLP with 2 layers and a hidden dimensionality 64 with batch normalization. The batch size was set to 256. Adam was used to train the network with a learning rate of 0.001, a weight decay of 5 × 10^–4^, and otherwise default parameters.

### 2.5 Data Sets for Studying the Validity and Utility of RF Kernel for Centered Kernel Alignment

The validity and utility of RF kernel for CKA were investigated with data from Therapeutics Data Commons (tdc).^47^ Specifically, five binary classification data sets for cytochrome P450 inhibition based on the work of Veith et al.^48^ and three binary classification data sets for cytochrome P450 substrates based on the work of Cheng et al.^49^ were used in this study.

### 2.6 Descriptors

All molecular descriptors employed in this work were generated with RDKit.^50^ They include the 206 physicochemical descriptors implemented in the software, except “Ipc” and “BertzCT” (as they produced double overflows for large molecules). The FAME fingerprints were generated with CDPKit^51^ as described in ref. (52).

### 2.7 Model Performance Metrics

The primary performance metric employed in this work is the Matthews Correlation Coefficient (MCC). The MCC is a measure of the quality of a binary classification model. It is in the interval [−1,1], with 1 indicating perfect classification performance and −1 indicating inverse prediction. It is defined as
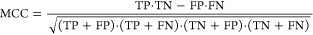
4where TP is the number of true positives, TN is the number of true negatives, FP is the number of false positives, and FN is the number of false negatives.^53^ The MCC is a reliable measure of the quality of a classification model because a high MCC is only achieved if the model performs well across all four confusion matrix categories.^54^

## 3 Results

### 3.1 Rotation-Invariant Learning Algorithms Can Generate Rotated Embeddings

Broadly speaking, a rotationally invariant learning algorithm is a model producing results that do not change with the rotation of the coordinate system (see Definition 1 in Section 2.1 for details and an example). Because MLPs are rotationally invariant learning algorithms,^10^ we expect the embeddings created during end-to-end training to be rotated. The dot product of two vectors is invariant to orthogonal transformations (i.e., rotation and reflection). In contrast, the RF kernel is not invariant to rotations because decision trees generate axis-aligned splits. Therefore, the decision boundary is not equivariant concerning feature rotation. In other words, it is to be expected that an RF used for calculating the RF kernel will not yield good classification performance when trained on embeddings obtained from training an MLP. Therefore, the RF kernel loses its ability to consider label information for partitioning adequately and, consequently, the ability to consider the label for CKA.

To show that the rotational invariance of MLP classifiers can lead to embeddings in end-to-end training whose features do not carry meaning individually (due to the occurrence of rotation), we calculated the CKA with linear and RF kernel and evaluated the performance of RFs on embeddings generated by convolutional encoders.

MNIST is a particularly suitable data set for showing that rotation can occur after a convolutional encoder because its simplicity enables RFs to produce good predictions on the raw images and because multiple representations of the hand-drawn digits are available: images,^41^ graphs (MNISTSuperpixel^42^), and sequences (MNISTStroke^43^). We employed three types of NNs to embed the three MNIST representations. Specifically, we considered MNIST a binary classification task, assigning all digits lower than five to the positive class. For the embedding of the images, we employed the ResNet18^12^ architecture, whereas for MNISTSuperpixel we employed a GIN.^14^ The sequences of MNISTStroke were encoded with an LSTM, summing over all outputs. An MLP was connected to each convolutional encoder and trained end-to-end.

We trained the NNs five times on the identical train-test split with different initializations. The use of the identical train-test split enables comparisons across seeds (otherwise, different samples would be compared with each other, leading to CKAs close to 0 because there is no more dependence between the samples). CKAs with the linear kernel and the RF kernel were calculated for all combinations of models and random seeds.

For all models, including the RF trained on the vectorized images, the MCC was above 0.8 (Table 1). In contrast, the performance of the RFs trained on the latent representations of the NNs was as good as a coin flip even though the training set was fit perfectly. These results indicate that the RF struggles with the NNs’ representation of the digits, likely due to the lack of invariance to the rotation of feature spaces.

**Table 1:** MCC of the Three NNs and an RF Trained on the Vectorized Images and Average MCC of the RFs (MCC_rf_) Used for Calculating the RF Kernel

Model	MCC	MCC_rf_
GIN	0.839 ± 0.003	0.024 ± 0.162
ResNet	0.990 ± 0.001	–0.011 ± 0.281
LSTM	0.964 ± 0.003	0.023 ± 0.103
RF	0.950 ± 0.001	-

**Figure 3:**
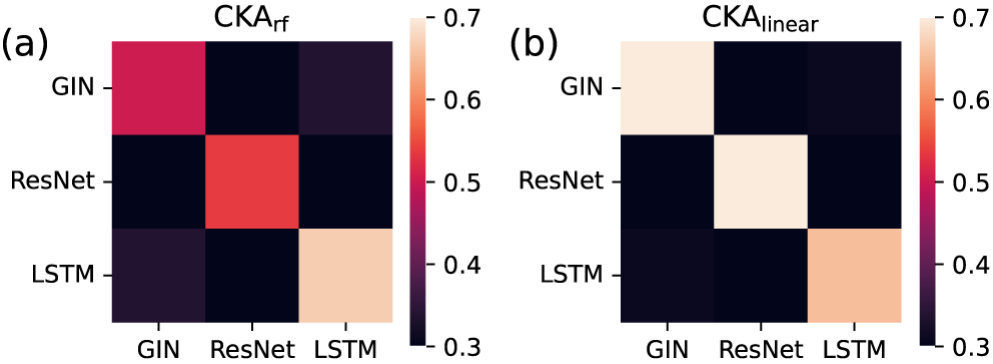
Comparison of the ability of the (a) RF kernel and (b) linear kernel to identify similarities between graph-based, sequence-based, and image-based representations of MNIST.

As shown in Figure 3, the linear kernel can better differentiate between representations of MNIST because the values along the main diagonal are larger and, hence, the difference to other representations is larger. This experiment demonstrates that CKA can differentiate between learned embeddings stemming from different encoding methods.

### 3.2 Centered Kernel Alignment with Random Forest Kernel Is the Natural Choice for Random Forests

Because RFs and MLPs are commonly used for predicting molecular properties, we next analyzed which kernel to use with CKA for a given model type. The literature argues that using a rotational invariant kernel for NNs based on MLPs is adequate because the convolutional encoder learns a representation that can be rotated in any direction and magnitude.^21,34^ Hence, CKA with the dot product as the kernel (CKA_linear_) is an established similarity measure for MLPs.^21−23^

**Figure 4:**
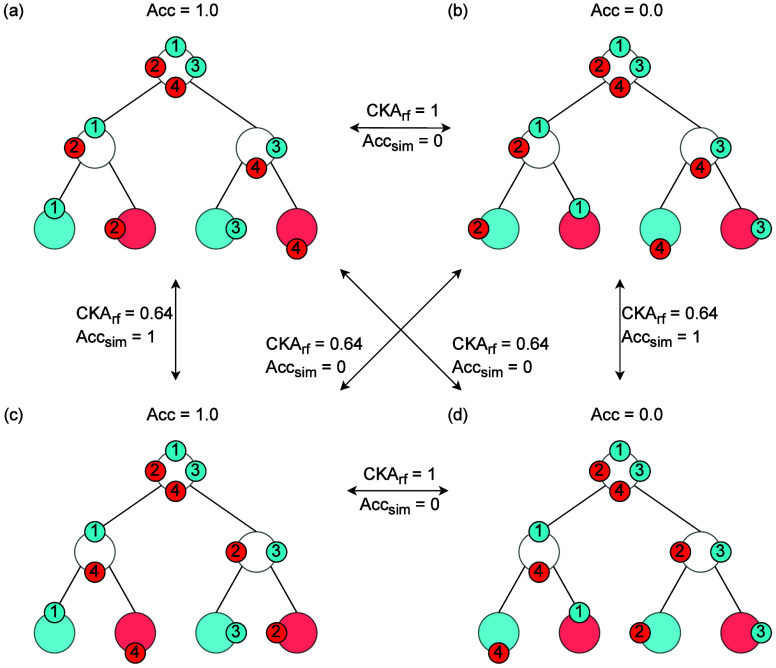
Displayed are four RFs, each composed of a single decision tree with four test samples (circles 1 to 4) in the corresponding partitions. The color of the samples indicates their label, whereas the color of the tree nodes indicates the predicted label.

In the previous section, we reported that the RF kernel is unsuitable for NNs due to its mismatched behavior regarding rotation. However, the RF kernel is a natural choice for RFs. This is because the RF kernel makes the internal representations of the RF available and because the behavior of the RF and the RF kernel concerning the data’s rotation is similar. Furthermore, CKA_rf_ has several advantages over pure performance similarity (Acc_sim_) assessment when comparing RFs based on distinct embedding strategies. In cases where two RFs obtain high values for Acc_sim_, CKA_rf_ may detect differences related to the rules according to which the RFs split the data. This case is illustrated in Figure 4 by the comparison of (a) with (c) and of (b) with (d). Likewise, CKA_rf_ will detect similarities in the structures of two RFs with distinct classification performances in cases where the rules derived from the training set generate embeddings that are perfectly aligned even though the predictions are wrong (Figure 4 (a) vs (b) and (c) vs (d)) Moreover, CKA_rf_ can identify similarities in the structure of two RFs even when they do not generalize to the test set (Figure 4 (b, d)). This is because the internal structure of the trees can be similar even for models with poor performance.

### 3.3 Tests with Multiple Data Sets Confirm Our Analysis of the Random Forest Kernel for Centered Kernel Alignment

To show that our arguments about CKA_rf_ being a good similarity measure for RF (see the preceding section) hold empirically, we trained RFs on eight data sets from tdc(47) to predict cytochrome P450 substrate and inhibition by small molecules. More specifically, we derived circular fingerprints of radius one to four (FP_*r*_ with ) and calculated Gram matrices based on the linear and RF kernels for each model. The Gram matrices were used to calculate the embedding similarity (CKA), and the intermodel performance was measured in terms of MCC (we call the MCC between two model predictions MCC_inter_). This procedure was performed using five random seeds.

**Figure 5:**
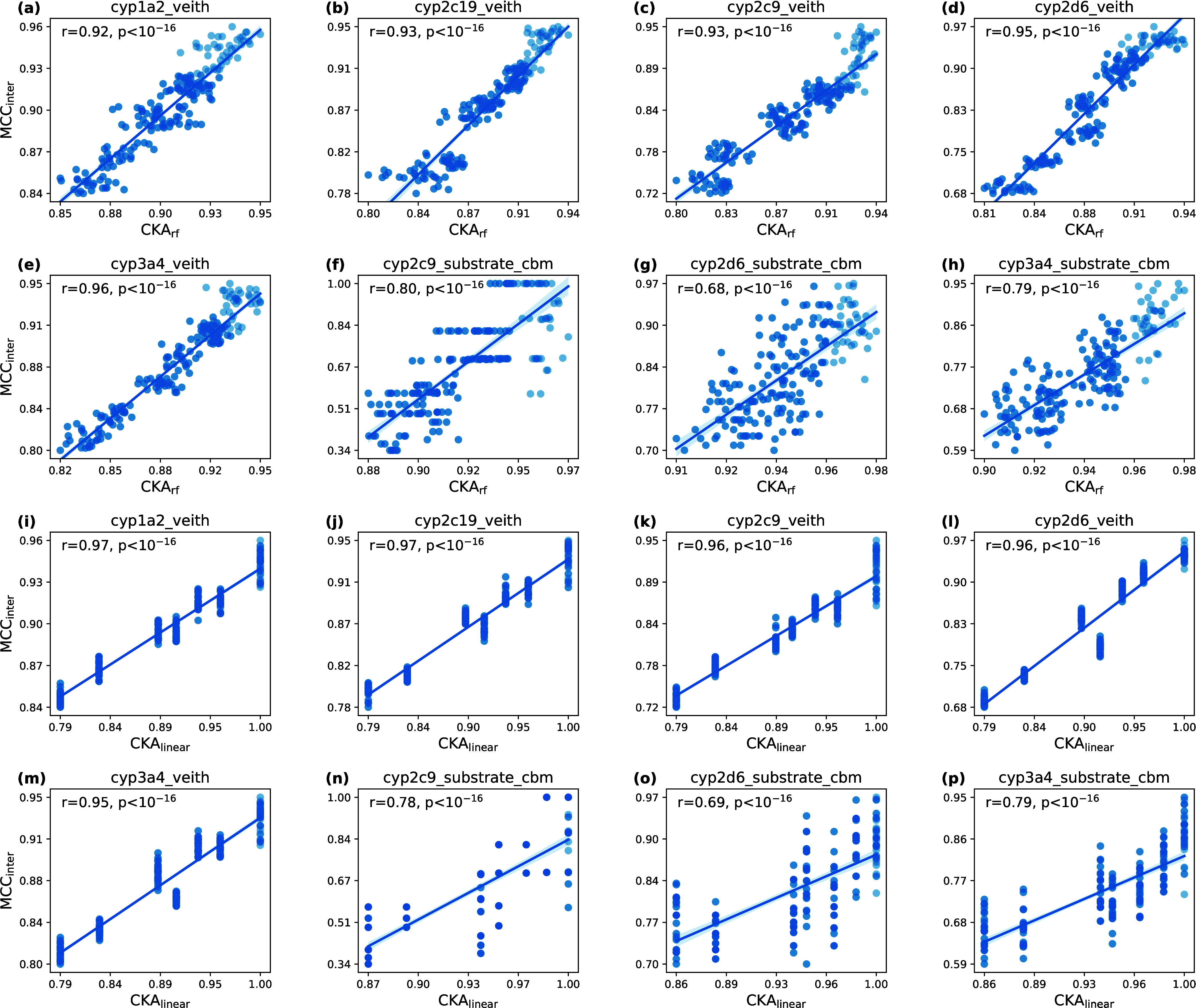
Pearson correlations *r* and corresponding *p* values calculated for the prediction similarities (MCC_inter_) and the embedding similarities of RFs with varying radii for the tdc data sets. Darker shades of blue indicate overlaps of data points.

The correlation between the embedding and performance similarity measures is shown in Figure 5. From the plots, we learn that the correlation between CKA_rf_ is comparable to CKA with linear kernel CKA_linear_ even though there is a sampling error in the calculation of the Gram matrices with RF kernel. However, the linear kernel exhibits discontinuities because of the binary nature of circular fingerprints, resulting in gaps in CKA_linear_. Furthermore, the plots in Figure 5 also highlight the central shortcomings of CKA_linear_ for RFs: differentiation between models with the same featurization strategy is impossible. Model agreement varied substantially for models with low prediction performance despite of the CKA_linear_ being the same (apparent as vertical series of data points in panels (n), (o), and (p)). On the other hand, CKA_rf_ differentiated model pairs with similar intermodel prediction (apparent as approximately horizontal series of data points, most prominent in panel (f)). Furthermore, the strong correlation observed for performance similarity and CKA_rf_ indicates that two RFs with similar predictions also have similar structures (panels (a) to (h)), giving a good intuition for interpretation of the similarity measure.

### 3.4 The Radii of Molecular Fingerprints Do Not Have a Substantial Impact on Model Performance

A recent study benchmarking circular fingerprints found no substantial impact of the applied fingerprint radius on similarity-based virtual screening.^4^ Furthermore, there is evidence that optimizing fingerprint hyperparameters does not lead to major RF performance improvements.^55^ Also in this work, we did not detect any substantial increases in model performance for cytochrome P450 substrate and inhibition prediction as we altered the fingerprint radii (Table 2). The impact was particularly small for well-performing models (i.e., models with MCC > 0.4).

**Table 2:** Average MCC Values with Standard Deviations (5 Random Seeds) for RFs Trained on tdc Data with Different Embeddings

	FP_1_^a^	FP_2_^a^	FP_3_^a^	FP_4_^a^	PC^b^
cyp1a2_veith	0.673 ± 0.004	0.669 ± 0.003	0.657 ± 0.004	0.654 ± 0.005	0.724 ± 0.005
cyp2c19_veith	0.618 ± 0.003	0.613 ± 0.002	0.615 ± 0.006	0.608 ± 0.006	0.632 ± 0.004
cyp2c9_veith	0.551 ± 0.006	0.560 ± 0.006	0.537 ± 0.008	0.516 ± 0.004	0.557 ± 0.006
cyp2d6_veith	0.492 ± 0.007	0.504 ± 0.007	0.485 ± 0.007	0.459 ± 0.002	0.457 ± 0.004
cyp3a4_veith	0.562 ± 0.001	0.557 ± 0.003	0.557 ± 0.005	0.558 ± 0.007	0.566 ± 0.007
cyp2c9_substrate_cbm	0.162 ± 0.050	0.253 ± 0.026	0.245 ± 0.000	0.187 ± 0.032	0.202 ± 0.037
cyp2d6_substrate_cbm	0.332 ± 0.024	0.226 ± 0.027	0.226 ± 0.009	0.230 ± 0.031	0.397 ± 0.029
cyp3a4_substrate_cbm	0.258 ± 0.013	0.236 ± 0.031	0.278 ± 0.013	0.251 ± 0.026	0.174 ± 0.017

a FP_*r*_ circular fingerprints of radius *r*.

b Physicochemical properties (see Section 2 for details).

**Figure 6:**
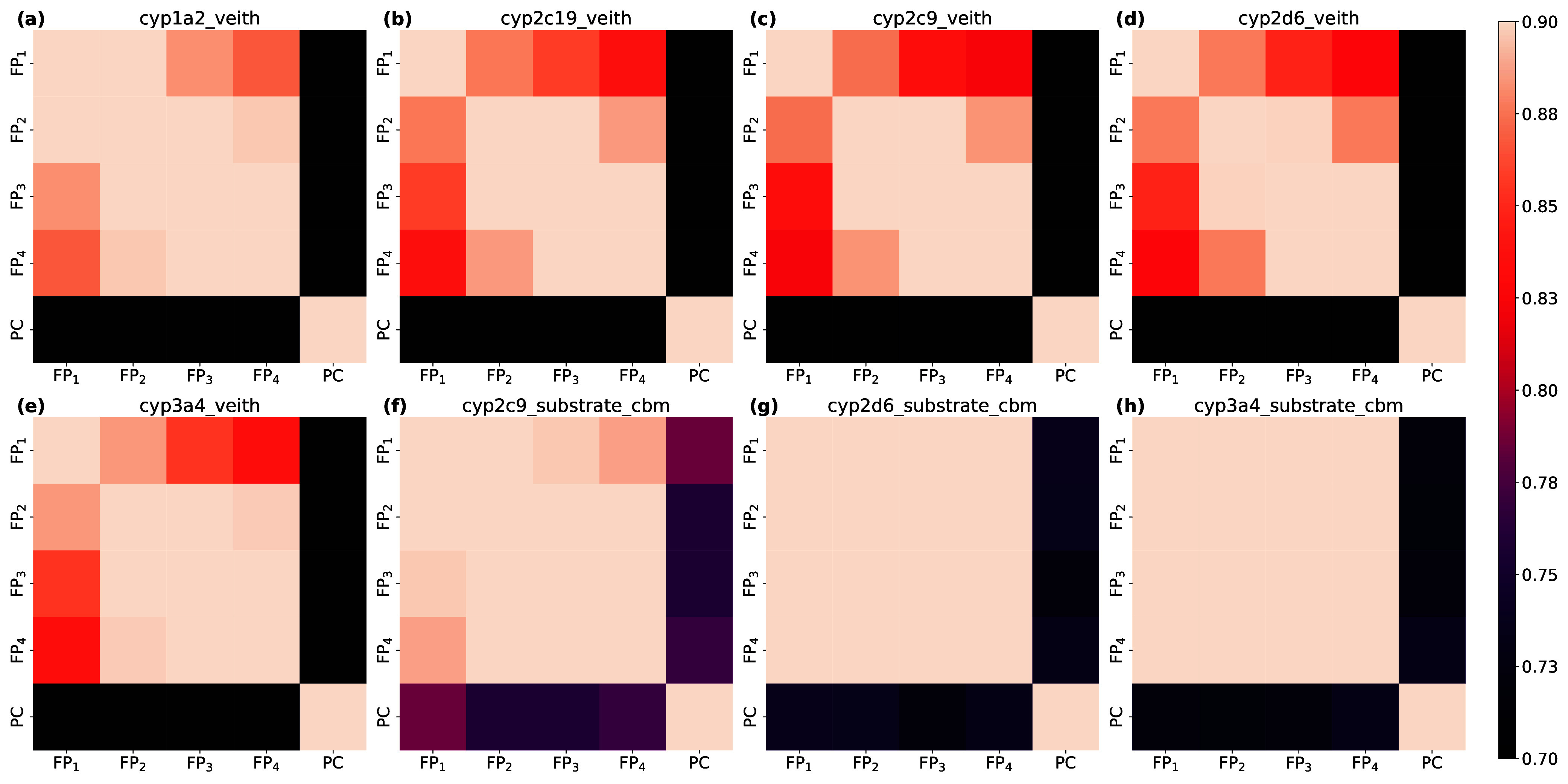
Average similarity of RFs with five different featurization strategies across five seeds. FP_r_ denotes the models using circular fingerprints with radius *r* as features; PC denotes the models using physicochemical properties. Notably, the circular fingerprints of radius 1 exhibited lower embedding similarities than any other combination of circular fingerprints. Panels (a) to (e) show the CKA_rf_ similarities of RF models generated from the cytochrome P450 inhibition data sets, whereas panels (f) to (h) show the similarities for the cytochrome P450 substrate data sets.

To understand the reason for the insensitivity of the models toward changes in the fingerprint radii, we employed CKA_rf_ to the RF models derived from circular fingerprints of radius one to four (FP_*r*_ with *r* ∈ {1,2,3,4}). In addition, we generated RF models from physicochemical properties (PC) to serve as a reference. A total of 200 models resulted from the combination of five RFs trained for each of the five embedding strategies and eight data sets. Across all data sets, we recorded high CKA_rf_ values between the embedding strategies of circular fingerprints of various lengths (Figure 6). In contrast, the CKA_rf_ between models with physicochemical properties and circular fingerprints was considerably lower. Even though the tasks vary in difficulty, the results were consistent due to CKA’s ability to detect similarities among models with distinct performance (discussed and experimentally shown in the previous sections). Based on this observation, we conclude that well-performing RFs do not show substantial performance improvements upon changes in the fingerprint radii because the learned decision rules largely remain unchanged.

### 3.5 Random Forest Decision Rules Remain Largely Unaltered When Training on Rooted Fingerprints Exceeding a Threshold Radius

FAME is a collection of RF models for site-of-metabolism (SoM) prediction.^52,56^ Like several related tools, FAME employs rooted fingerprints for describing the atom environment of SoMs and non-SoMs. Previously, we showed that the fingerprint radius does not substantially impact the performance of FAME. One reason for this behavior is the fact that the physicochemical descriptions included in the FAME fingerprint implicitly describe larger environments.^52,56^

**Figure 7:**
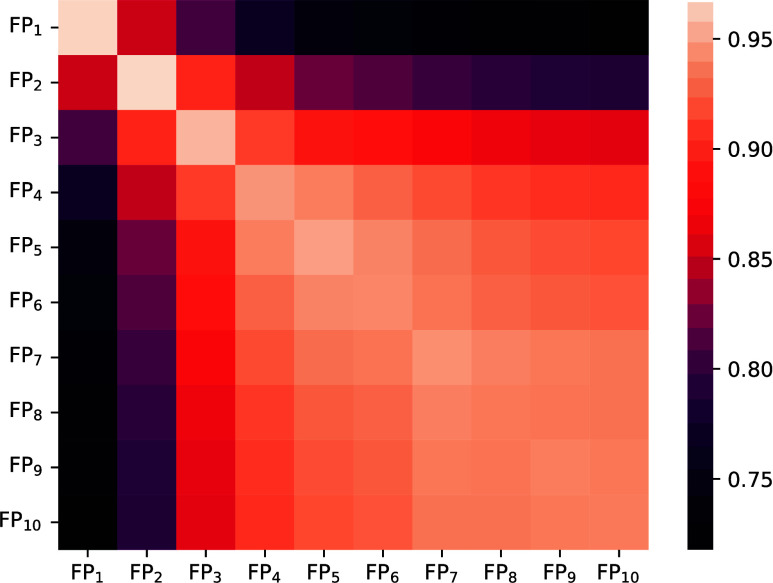
CKA_rf_ (embedding similarity) between RFs with FAME fingerprint with varying fingerprint radii, averaged over five random seeds.

As the radius of the rooted fingerprint increases, we assume that the learned decision rules become increasingly similar. This should become apparent at a radius threshold r* beyond which only a few atoms feature path lengths of at least r*. To confirm this assumption, we calculated the CKA_rf_ between FAME RF models with fingerprint radii between 1 and 10 (FP_*r*_). As shown in Figure 7, a gradual change of similarity can be observed for models as the radius is increased from 1 to 4. However, models derived from fingerprints with radii above 4 show high similarity, indicating that r* is likely between 4 and 7, confirming our assumption. Additionally, this experiment confirms the robustness of CKA_rf_ because the number of bits of the FAME fingerprints changes with the radius.

## 4 Conclusions

In this work, we present the adaptation of CKA for use with RFs. More specifically, we combine CKA with the RF kernel (CKA_rf_) to ensure compatibility of the similarity measure and learning algorithm and make the RF’s internal data representations available. CKA_rf_ enables, e.g., the differentiation of RFs with similar prediction performance. We validate the technique by demonstrating its correlation with performance similarity. Employing CKA_rf_ to molecular embeddings, we show that the radius of circular fingerprints has no substantial effect on the performance of RFs because the rules learned by the forests are similar. Furthermore, we use CKA_rf_ to determine the radius of fingerprints beyond which only a few new decision rules are learned for SoM prediction, showing the robustness of CKA_rf_ even in cases where different feature vector lengths are used.

## Data Availability

All data used in this work was sourced from tdc,^47^torch,^44^torchGeometric,^45^ or ref. (43) and is accessible via these software libraries’ Python APIs. The source code of the approach presented in this work is available from https://github.com/MatthiasWel/RFkernel/.
